# Hydrogel-hydroxyapatite-monomeric collagen type-I scaffold with low-frequency electromagnetic field treatment enhances osteochondral repair in rabbits

**DOI:** 10.1186/s13287-021-02638-6

**Published:** 2021-11-13

**Authors:** Jiyuan Yan, Chaoxu Liu, Chang Tu, Ruizhuo Zhang, Xiangyu Tang, Hao Li, Huaixi Wang, Yongzhuang Ma, Yingchi Zhang, Hua Wu, Gaohong Sheng

**Affiliations:** 1 Department of Orthopedics, Tongji Hospital, Tongji Medical College, Huazhong University of Science and Technology, Jiefang Avenue 1095, Wuhan, 430030 Hubei People’s Republic of China; 2 Department of Orthopedics, Renmin Hospital of Wuhan University, Wuhan, Hubei People’s Republic of China; 3 Department of Radiology, Tongji Hospital, Tongji Medical College, Huazhong University of Science and Technology, Wuhan, Hubei People’s Republic of China; 4 Department of Spine and Spinal Cord Surgery, Henan Provincial People’s Hospital, People’s Hospital of Zhengzhou University, Henan, Zhengzhou People’s Republic of China; 5 Department of Orthopedics, Shanxi Bethune Hospital, Taiyuan, Shanxi People’s Republic of China; 6 Department of Traumatology, Tongji Hospital, Tongji Medical College, Huazhong University of Science and Technology, Jiefang Avenue 1095, Wuhan, 430030 Hubei People’s Republic of China

**Keywords:** Hydrogel, Hydroxyapatite, Monomeric Collagen type I (Col1), Electromagnetic fields, Osteochondral defects, Mesenchymal stem cells

## Abstract

**Background:**

Cartilage damage is a common medical issue in clinical practice. Complete cartilage repair remains a significant challenge owing to the inferior quality of regenerative tissue. Safe and non-invasive magnetic therapy combined with tissue engineering to repair cartilage may be a promising breakthrough.

**Methods:**

In this study, a composite scaffold made of Hydroxyapatite-Collagen type-I (HAC) and PLGA-PEG-PLGA thermogel was produced to match the cartilage and subchondral layers in osteochondral defects, respectively. Bone marrow mesenchymal stem cells (BMSC) encapsulated in the thermogel were stimulated by an electromagnetic field (EMF). Effect of EMF on the proliferation and chondrogenic differentiation potential was evaluated in vitro. 4 mm femoral condyle defect was constructed in rabbits. The scaffolds loaded with BMSCs were implanted into the defects with or without EMF treatment. Effects of the combination treatment of the EMF and composite scaffold on rabbit osteochondral defect was detected in vivo.

**Results:**

In vitro experiments showed that EMF could promote proliferation and chondrogenic differentiation of BMSCs partly by activating the PI3K/AKT/mTOR and Wnt1/LRP6/β-catenin signaling pathway. In vivo results further confirmed that the scaffold with EMF enhances the repair of osteochondral defects in rabbits, and, in particular, cartilage repair.

**Conclusion:**

Hydrogel-Hydroxyapatite-Monomeric Collagen type-I scaffold with low-frequency EMF treatment has the potential to enhance osteochondral repair.

**Supplementary Information:**

The online version contains supplementary material available at 10.1186/s13287-021-02638-6.

## Introduction

Articular cartilage is mainly composed of chondrocytes and the extracellular matrix (ECM), such as aggrecan and collagen type 2 (Col2) with complex hierarchical structure [1]. During daily activities, cartilage plays an important role in the transmission of joint loads and lubrication of articular movement [2]. Cartilage damage is a common medical issue caused by trauma, aging, and disorders of cartilage itself and subchondral bone (including osteoarthritis, osteonecrosis, and osteochondritis dissecans) [3, 4]. The chronic pain and dysfunction of affected joints significantly diminish the patient’s life quality. Owing to the avascularity of cartilage and low metabolic activity of chondrocytes, such cartilage defects have limited capacity to heal spontaneously [5, 6]. Several therapeutic strategies have been developed to treat cartilage damage, including bone marrow stimulation (such as microfracture and subchondral bone drilling) [7, 8], autologous chondrocyte implantation [9–11] and autografts transplantation [12, 13]. However, the complete repair of hyaline cartilage remains a major challenge in clinical practice owing to the inferior quality of regenerative tissue [14, 15]. Moreover, the results of long-term follow-up have revealed a poor clinical prognosis in patients with cartilage defects because of cartilage degradation, regardless of the treatment therapy [16–19].

Recently, tissue engineering has provided novel insights into the treatment of osteochondral defects [20, 21]. Among various biomaterials, injectable thermogels have received significant attention owing to several unique advantages, including minimally invasive implantation, sterilization by filtration, no need for organic solvents, and good plasticity for irregularly shaped defects or cavities [22]. Generally, drugs or cells are easily mixed and encapsulated in aqueous solution (sol state) at ambient temperature. Then, the solution undergoes sol-to-gel transition to form gels when heated. This in situ thermogelling system has been widely applied, for example, in drug delivery, prevention of postoperative adhesion, transcatheter arterial embolization, and tissue repair [22–24]. For instance, thermosensitive hydrogel based on poly(lactide-co-glycolide)-poly(ethylene glycol)-poly(lactide-co-glycolide) (PLGA–PEG–PLGA) triblock copolymer are extensively applied in clinical use owing to its injectability, excellent biocompatibility, and biodegradability [25, 26]. Furthermore, a study indicated that this thermogel performed better than fibrin gel with regard to relieving symptoms of rheumatoid arthritis (RA) using bone marraw mesenchymal stem cell (BMSC) culture [27]. In view of cartilage tissue engineering, the PLGA–PEG–PLGA copolymer simulates a 3D network structure, similar to that of native cartilage, to support proliferation and differentiation of BMSCs. Furthermore, the main product of poly(lactide-co-glycolide) (PLGA) degradation is glutamate, which is the most abundant amino acid in articular cartilage [28]. Cell-based tissue engineering is currently a common and effective therapy for cartilage regeneration. Among the numerous cell types, mesenchymal stem cells (MSCs)––BMSCs, in particular––are apparently the most promising candidates owing to their capacity of self-renewal, proliferation, and multi-lineage differentiation [29, 30].

Remarkably, increasing evidence has demonstrated that physical stimuli such as electromagnetic fields (EMF) could promote chondrogenic differentiation of BMSCs [31]. The EMF exposure induced chondrocyte proliferation and differentiation, and ECM synthesis by variable potential mechanisms, including regulating calcium channel in cell membrane [32, 33], increasing transforming growth factor beta (TGFβ) and bone morphogenetic protein 2 (BMP2) expressions [34, 35], and modulating MSCs secretome and paracrine function [36]. Stefani et al. [37] reported that EMF stimulation improved repair and integration of engineered constructs in an animal model. In addition, EMF was suggested to exert an anti-inflammatory effect through improving immunomodulatory function of MSCs [38, 39]. Notably, another advantage was that EMF could significantly expedite osteogenesis of MSCs and bone regeneration [40, 41]. The reconstruction of subchondral bone is critical to cartilage restoration, as cartilage defects frequently involve destruction of the subchondral bone. Thus, EMF has attracted considerable attention for repairing osteochondral defects with engineered constructs.

In this study, we explored the effect of sinusoidal EMF (SEMF) on osteochondral repair combined with BMSC-based tissue engineering, especially cartilage regeneration, using the PLGA-PEG-PLGA thermogel, and its underlying molecular mechanisms. According to our previous study, the parameters of EMF (15 Hz, 1 mT, 4 h/day) could efficiently facilitate proliferation and chondrogenic differentiation of BMSCs in vitro [42]. After exposure to EMF for 7 and 14 days, the cultured BMSCs were harvested to analyze expression of targeted genes and proteins, respectively. The proliferation rate of BMSCs was also detected by cell counting kit-8 (CCK-8). In the in vivo experiments, the BMSCs-loaded thermogels with or without EMF pretreatment were first injected on HAC scaffolds at ambient temperature and incubated at 37 °C for 30 min. The prepared composite scaffolds were then implanted in osteochondral defect regions. After 12 weeks post implantation, histological and immunohistochemical analyses were performed to evaluate regenerated tissue. Meanwhile, subchondral bone was also assessed by micro-CT. These in vitro and in vivo results suggest that EMF has great potential to enhance osteochondral repair, especially cartilage regeneration; this provides the basis for further research and clinical translation of EMF for the repair of cartilage defects.

## Materials and methods

### Preparation and characterization of PLGA–PEG–PLGA thermogel

PEG, LA, GA, and Sn(Oct)_2_ were purchased from Sigma-Aldrich (St. Louis, MO, USA). As reported in a previous study [43], the PLGA–PEG–PLGA copolymer was synthesized by ring-opening polymerization approach. Poly(ethylene glycol) (PEG) (1500 g/mol, 60 g) was dried under vacuum in a three-necked flask as the macroinitiator. Lactide (113.46 g) and glycolide (30.48 g) were added to the flask and mixed. Deuterated chloroform with the internal standard tetramethylsilane was used as the solvent to dissolve the mixture of PEG, lactide and glycolide. The concentration of the PLGA-PEG-PLGA polymer is 20 wt %. After incubation of the mixture at 80 °C for 30 min, the reaction was activated by the catalyst stannous octoate (Sn(Oct)_2_) (0.08 g) at ~ 150 °C. Subsequently, the unreacted monomers were removed by cooling down to 120 °C with stirring for 3 h under vacuum, and the crude copolymers were then washed in 80 °C water three times to further eliminate water-soluble oligomers and unreacted monomers. For each washing, 6 ml of water was added to every 1 g of the copolymers. After purification and drying, the copolymers were harvested and stored at − 20 °C. We also measured the sol–gel transition temperature via tube inverting method. The aqueous solution of copolymer (0.5 mL, 25 wt%) was placed into a 2 mL centrifuge tube at 4 °C. Then, the tube was gradually heated in a water bath with a temperature increment of 1 °C per step. After equilibration at each temperature, the tube was inverted to observe the liquidity of copolymer solution. When there was no evidence of flow for at least 30 s, the temperature was recorded and determined as the sol–gel transition temperature.

### Preparation and characterization of HAC scaffold

The porous scaffolds were manufactured from hydroxyapatite (HA) and monomeric Collagen type I (Col1) in accordance with our previous study [44]. The collagen (3 g) was mixed with HA (2.5 g) that had been dissolved in HCl (0.525 mol/L, 4.5L) to obtain a uniform HAC solution. By adjusting the pH value and temperature, the HAC solution would be collected after centrifugation and freeze drying. The produced scaffolds were cylindrical with a diameter of 4 mm and height of 5 mm. In addition, we observed the microstructure of scaffolds via scanning electric microscopy (SEM; TESCAN VEGA 3, Brno, Czech Republic) and then analyzed their porosity, pore size, and compression strength.

### EMF device and exposure

**Fig. 1:**
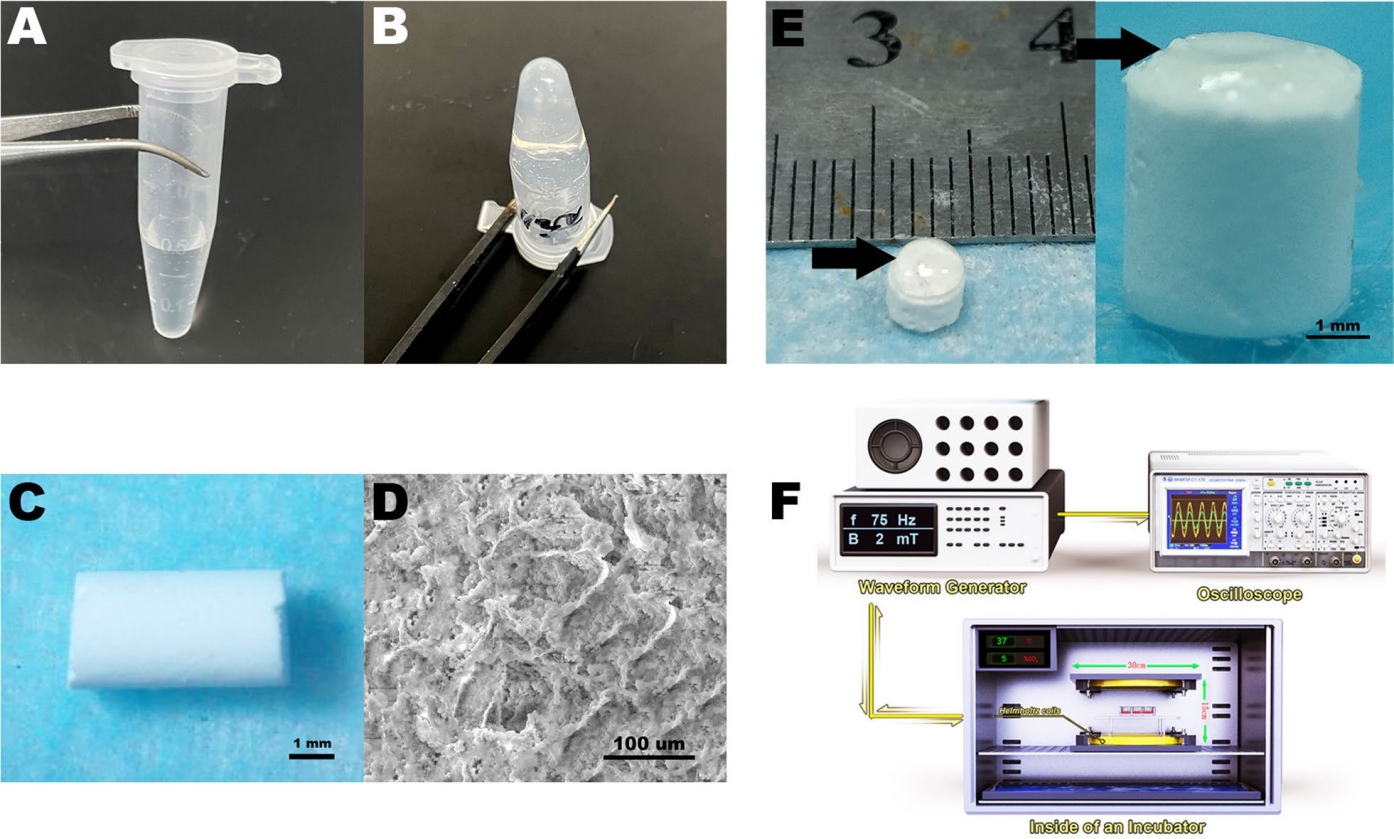
Characterization of composite scaffold and schematic diagram of EMF device. The components of Thermo-sensitive PLGA-PEG-PLGA are Poly ethylene glycol (PEG), dl-Lactide (LA) and glycolide (GA). The molecular weight of PEG is 1500. LA (113.46 g), GA (30.48 g), PEG (60 g) were reacted in the solvent of Deuterated chloroform with the internal standard tetramethylsilane. **A** Thermo-sensitive PLGA–PEG–PLGA copolymers exhibited a free-flowing sol at ambient temperature (25 °C) and **B** spontaneously converted into a semi-solid hydrogel at body temperature (36 °C). Every 100 g of HAC scaffold contains 45 g of HA and 55 g of collagen. **C** Macroscopic observation of cylindrical HAC scaffold produced by hydroxyapatite and collagen I. **D** Image of scanning electron micrograph (SEM) showing porous microstructure of HAC. **E** Assembly of composite scaffold. The cell-loaded aqueous sol was injected on the top of HAC and then incubated at 37 °C for 30 min. Arrow: the site BMSCs loaded. **F** Presentation of electromagnetic fields (EMF) device consisting of waveform generator, oscilloscope, and Helmholtz coils. Cells were stimulated at the center of coils, which was placed in an incubator at 37 °C and 5% CO_2_

The EMF device has been described in our previous studies [41]. The equipment was designed and manufactured by the Naval Engineering University of China. The signals produced by a waveform generator were amplified through an amplifier and transmitted to the coils. An oscilloscope was used to observe and verify the frequency and intensity of signals. The layout diagram of EMF facility is shown in Fig. 1F. On the basis of previous findings, we used a continuous EMF stimulation with parameters of 1 mT, 15 Hz, and 4 h/day in this study [45]. The culture plates were placed in the center of Helmholtz coils, which were located in a humidified incubator with 5% CO_2_ at 37 °C. The sham control samples were kept in another incubator under the same conditions without EMF exposure.

### Isolation and culture of rabbit BMSCs

BMSCs were isolated from iliac crest of New Zealand White rabbits. All procedures on animals were performed with approval from the Ethics and Animal Research Committee of Huazhong University of Science and Technology. The bone marrow and MSCs were harvested as described in the literature [46]. After anesthesia, bone marrow was aspirated via a small incision using an 18G needle and stored in a pre-heparinized syringe. Dulbecco’s modified Eagle’s medium (DMEM, Gibco), supplemented with 10% fetal bovine serum (FBS) and 1% penicillin and streptomycin, was added to marrow suspension. The isolated cells were cultured to adhere to the flask in a humidified incubator with 5% CO_2_ at 37 °C. The culture media was first changed after five days and refreshed every three days thereafter. Adherent cells were detached and passaged when they reached 80% confluence. Third passage BMSCs were used in the subsequent experiments.

### Cell culture in PLGA-PEG-PLGA thermogel and cell viability assay

After detachment and centrifugation, the rabbit BMSCs were resuspended in the PLGA-PEG-PLGA copolymer solution with a final concentration of 1.0 × 10^6^/mL. Subsequently, encapsulated BMSCs were placed in an incubator at 37 °C for 30 min to achieve sol-to-gel transition. The expansion medium was added on the surface of gel and replaced by fresh medium every three days. The cell-encapsulated gels were prepared for following experiments. To text proliferation, BMSCs were seeded into 96-well culture plates at a density of 1 × 10^4^ cells per well in DMEM medium and incubated for 24 h, and the viability of BMSCs were assessed using a cell counting kit-8 (CCK-8, Boster, Wuhan, China) according to the standard protocol. BMSCs were seeded in thermogels and cultured for 1, 4, 7, 10, and 14 days. Then, a total of 180 $\upmu$L culture medium, supplemented with 20 µL CCK-8 solution, was added to each well in a 96-well plate. After incubation for 1.5 h at 37 °C, the absorbance was read at 450 nm by a microplate reader (Bio-Rad, Richmond, CA, United States).

### Quantitative reverse transcription-polymerase chain reaction (qRT-PCR)

Total RNA from in vitro cultured BMSCs was extracted using the EZNA Total RNA kit (Omega Bio-Tek, USA) and first strand cDNA was synthesized from 2 $\upmu$g RNA with the Reverse Transcription kit (Toyobo Life Science, Japan). The SYBR Green Real-Time PCR Master Mix (Toyobo Life Science, Japan) was used to quantify the expression of target genes on CFX96 (Bio-Rad Laboratories, USA) system. The relative expression levels of genes were normalized to corresponding GAPDH and analyzed by 2^−ΔΔCt^ method. The sequences of primers used in this study were listed in Additional file 1: Table S1. All the experiments were performed in triplicates.

### Western blot

The total protein was extracted by radio immunoprecipitation assay lysis buffer (RIPA) supplemented with 1% protease and phosphatase inhibitors (Boster, Wuhan, China). The protein concentration was determined with a Bicinchoninic Acid Assay (BCA) protein assay kit (Boster, Wuhan, China). Then, 20 $\upmu$g protein samples were separated on 10% SDS-PAGE and transferred onto PVDF membranes (Millipore, USA). First, the membranes were blocked for 1 h with 5% bovine serum albumin (BSA) at 37℃. Second, membranes were incubated with cyclin D1, CDK4, PCNA, p-PI3K, PI3K, p-Akt, Akt, p-mTOR, mTOR, Col2, Aggrecan, SOX9, Wnt1, LRP6, β-catenin (all 1:1000) (Cell Signaling Technology, Danvers, MA) and GAPDH (1:400) (Boster, Wuhan, China) primary antibodies at 4 °C overnight and subsequently washed for three times with Tris-buffered saline with 0.1% Tween-20 (TBST). Then, membranes were incubated with horseradish peroxidase (HRP)-conjugated secondary antibodies (1:5000) (Boster, Wuhan, China) at room temperature for 1 h. Finally, the protein bands were visualized under the Western enhanced chemiluminescence (ECL) system (Thermo Fisher Scientific, USA). The corresponding GAPDH bands were served as loading control and the amount of target proteins were semi-quantified by Image Lab system version 5.1 (Bio-Rad Laboratories, USA). All western blots were independently repeated three times, and the representative bands were obtained.

### Immunofluorescence

The BMSCs were cultured in 24-well plates with a density of 5 × 10^5^ per plate in DMEM medium and incubated for 24 h. Then the culture medium was removed and cells was washed three times with PBS. Then, cells were fixed with 4% paraformaldehyde for 15 min, permeabilized with 0.5% Triton X-100 for 15 min, blocked with goat serum (the same species as secondary antibody) for 30 min at room temperature. In between these steps, cells were rinsed three times with PBS. Cells were incubated with anti- Col2, Aggrecan and SOX9 (1:200) (Cell Signaling Technology, Danvers, MA) primary antibodies with appropriate dilution at 4 °C overnight, followed by fluorescein labeled secondary antibodies (1:200) (Boster, Wuhan, China) at room temperature for 1 h in dark. Additionally, nuclei were counterstained by DAPI. Fluorescent microscopy (EVOS FL Auto Imaging System, Life technologies, Gaithersburg, MD) was used to observe fluorescence and acquire images.

### Flow cytometry analysis

BMSCs in the hydrogel were prepared into single cell suspension, incubated with anti- CD81 and CD90 (1:50) (R&D Systems, MN) primary antibodies for 45 min, and stained with secondary antibodies (1:100) (Boster, Wuhan, China) for 30 min at 4 °C. For each sample, 1 × 10^4^ cells were used for flow cytometry analysis. The corresponding isotype antibodies were used as negative control. The detection was performed by a flow cytometer (BD Biosciences, San Jose, CA, USA) and data analyzed by FlowJo software (FlowJo, LLC., Ashland, OR, USA).

### Rabbit model with femoral condyle defect and scaffold implantation

A total of 80 New Zealand rabbits (male, six-month-old) were used in this study. After anesthetization by intraperitoneal injection with 3% pentobarbital, the lateral femoral condyles were exposed and a 4 mm wide and 5 mm deep defect hole was created using a trephine [47, 48]. The different sterile constructs that had been prepared were then implanted into the punch defects. The incisions were carefully sutured layer by layer. The rabbits were sacrificed at 4- and 12-weeks post operation for the following experiments. The animals were randomly divided into five groups, and each group consisted of eight rabbits: (1) “Blank” group, without construct implantation; (2) “HAC” group, HAC scaffold without thermogel; (3) “HAC + gel” group, HAC scaffold and acellular thermogel; (4) “HAC + gel + MSC” group, HAC scaffold and BMSC-loaded thermogel; (5) “HAC + gel + MSC + EMF” group, HAC scaffold and BMSC-loaded thermogel, where the BMSCs had been stimulated by EMF. For the HAC scaffold, 100 g of HAC scaffold contains 45 g of HA and 55 g of collagen. For the HAC + gel, the ratio of HAC to gel is 5:1, the gel we used was PLGA-PEG-PLGA polymer. The size of HAC + gel is 4 mm in width and 5 mm depth, 1 × 10^6^ BMSCs were added into HAC + gel scaffold. The density of the BMSCs was 2 × 10^6^ cells per 100 µl of hydrogel. The EMF parameter was 1 mT, 15 Hz, and 4 h/day.

### Biomechanical properties of repaired osteochondral tissue

An Instron 5566 electromechanical testing device (Instron Corporation, USA) was applied to evaluate the biomechanical properties of regenerative osteochondral tissue at 12 weeks postoperatively. Repaired osteochondral samples with a thickness of 10 mm were cut from femoral condyle and then fixed on the plate. We used a custom stainless-steel rod (diameter = 4 mm) to compress our samples with a strain rate of 0.01 mm/s. The maximum displacement was set at 0.5 mm. The data recorded by a computer in real time were plotted as strain vs stress to capture the response of repaired osteochondral tissue under incremental load. The equilibrium Young’s modulus [49, 50] was also calculated through such unconfined compression test at 5% axial strain.

### Microcomputed tomography (micro-CT) analysis

The rabbits (*n* = 6) were euthanized to harvest specimens at 4 and 12 weeks after operation. The samples were then fixed for 2 days in 4% paraformaldehyde and scanned with micro-CT (Scanco, Brüttisellen, Switzerland) following standard protocol (70 kV, 114 $\upmu$A, and 20 $\upmu$m) [51]. Initial binary images were reconstructed to obtain 3D images using the built-in software. The bone mineral density (BMD) and the ratio of bone volume to total volume (BV/TV) within target regions were quantitatively evaluated.

### Histological evaluation

The rabbits (*n* = 6) were sacrificed at 4- and 12-weeks post operation, and femurs were harvested. Specimens were fixed in 4% paraformaldehyde solution for two days. After fixation, samples were decalcified and embedded in paraffin blocks. Then, 5 $\upmu$m thick slices were produced for subsequent staining (including hematoxylin and eosin (H&E), toluidine blue (T-B), safranin-fast green (S-F), and Masson's trichrome (M-T)) to observe new-formed tissues. The histological scores were blindly and independently evaluated by three authors (Gaohong Sheng, JiYuan Yan and Hao Li) based on a well-established histological scoring system (Additional files 1 and 2: Table S1 and S2) for osteochondral defects [52].

We also performed immunohistochemical (IHC) staining for chondrogenesis- and osteogenesis- related proteins, including Col2, aggrecan, Col X, Col1, and osteocalcin (OCN). The sections were immersed with 3% (w/v) H_2_O_2_ and blocked with 5% (w/v) BSA. After enzymatic antigen retrieval, sections were stained by primary antibodies at 4 °C overnight, followed by incubation of horseradish peroxidase (HRP)-conjugated secondary antibodies at room temperature for 50 min. The newly prepared 3, 3-diaminobenzidiine tetrahydrochloride (DAB) solution was added for visualization. Nuclei were counterstained with hematoxylin for 3 min.

### Statistical analysis

All quantitative data were presented as mean ± standard deviation (SD), unless otherwise specified, and ordinal data were shown as median (inter quartile range). For comparison between two groups, the Student’s *t* test was applied, while the statistical differences among groups were assessed through one-way analysis of variance (ANOVA) followed by the Tukey honestly significant difference (HSD) test. If equal variances are not assumed, Dunnett’s test was used. Differences among multiple groups over multiple times were evaluated by two-way ANOVA followed by the Bonferroni’s post-hoc tests. In terms of ordinal data like histological scores, the Kruskal–Wallis test was used to analyze differences. A value of *p* < 0.05 was considered statistically significant. SPSS 20.0 software (IMB Corp., USA) was used to perform statistical analyses.

## Results

### Characterization of HAC scaffold and PLGA-PEG-PLGA thermogel

As indicated in Fig. 1A, PLGA–PEG–PLGA copolymer appeared to be aqueous solution at 4 °C and converted into gel state without fluidity when heated up to 37 °C (Fig. 1B). Moreover, the sol–gel transition temperature of the copolymer was measured as 36 °C. The fabricated HAC scaffold presented a cylindrical shape with a diameter of 4 mm and height of 5 mm (Fig. 1C). The microstructure and morphology were further observed via SEM, and the images showed evenly distributed pores (Fig. 1D). The mean porosity was calculated as 77 ± 5.79%, and the compression modulus was 13.46 ± 0.88 MPa. Given such a porous feature of HAC scaffold, the PLGA-PEG-PLGA aqueous solution could penetrate the scaffold at a relatively low temperature and be firmly adhered to HAC after completing sol–gel transition at 36 °C (Fig. 1E).

### PLGA–PEG–PLGA hydrogel showed a good biocompatibility

**Fig. 2:**
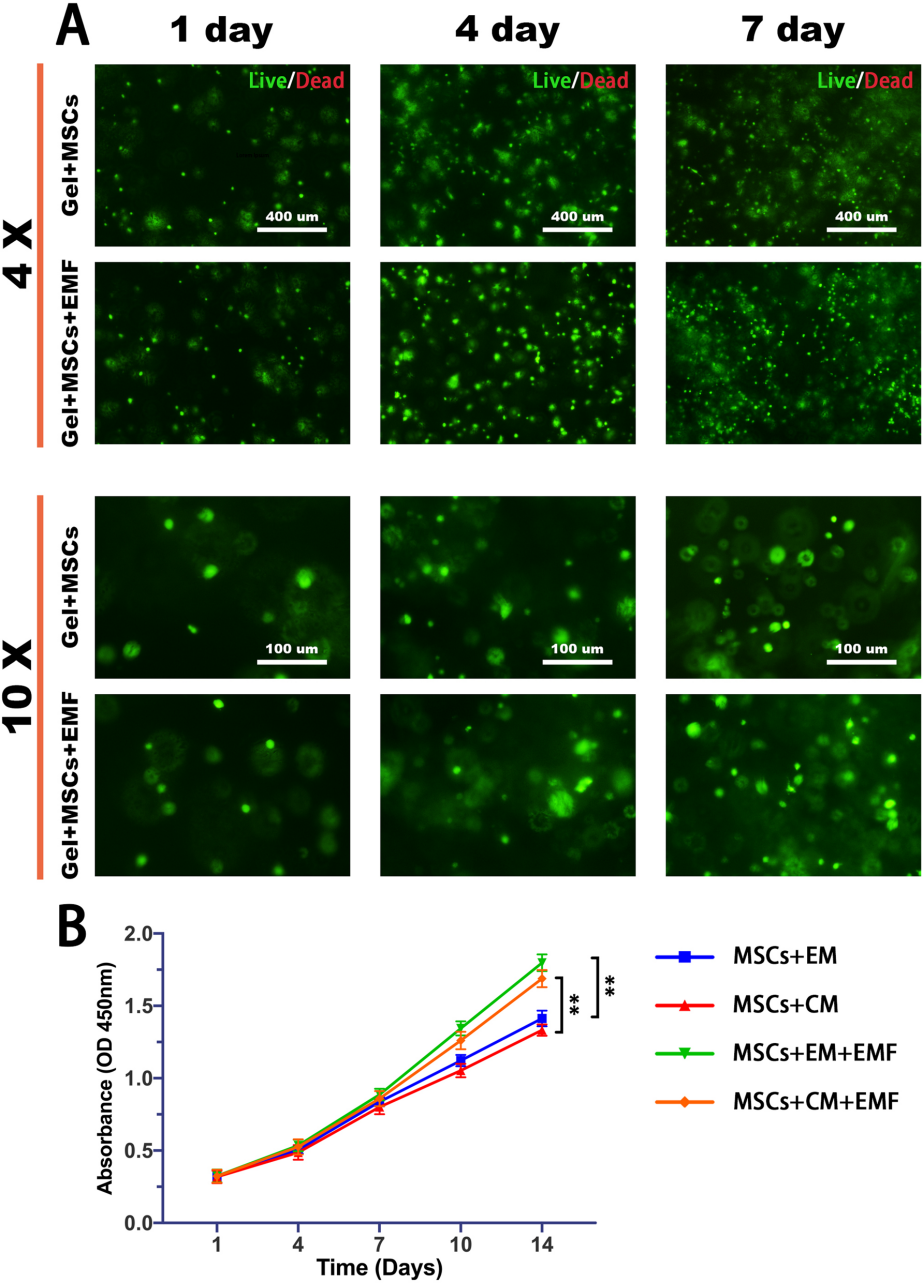
Cell culture and proliferation in hydrogel with or without EMF. **A** Images of Live/Dead staining demonstrate that the hydrogel supports cell viability and expansion of BMSCs after culturing for one, four, and seven days (green: live cells, red: dead cells). The cells were evenly distributed in the gel. **(B)** BMSCs proliferated over time, and EMF could significantly promote cell proliferation both in expansion medium (EM) and chondrogenic medium (CM). Data are shown as mean ± standard deviation (*n* = 5). **#** indicates no significant difference, *p < 0.05, **p < 0.01, ***p < 0.001 compared to group without EMF

The PLGA-PEG-PLGA hydrogel provided a three-dimensional environment for cell growth, which is distinct from the monolayer cell culture. Further, the interaction between cell and hydrogel matrix was weakened owing to the hydrophobicity of PEG chain segment. As shown in Fig. 2A, BMSCs within hydrogel presented a spherical morphology, while cells growing on plate with flat surface exhibited a typical spindle shape. The BMSCs were cultured in PLGA–PEG–PLGA hydrogel for live/dead staining at 1, 4, and 7 days. The results showed that hydrogel did not affect activity and proliferation of BMSCs. We also found the cells were fairly evenly distributed in hydrogel.

### EMF promotes proliferation of BMSCs partly by activating PI3K/AKT/mTOR pathway

Live/dead staining revealed that the cell number in EMF group appeared to increase compared with control group, especially at Day 7 (Fig. 2A). To further assay the effect of EMF on cell proliferation, CCK-8 was performed at multiple points for a two-week period (Fig. 2B). The results showed that BMSCs proliferated over time in each group. Furthermore, there was no significant difference among groups in cell number within 1 week of EMF induction. However, EMF could significantly promote proliferation of BMSCs after stimulation for over ten days both in expansion medium (EM) and chondrogenic medium (CM) groups.

**Fig. 3:**
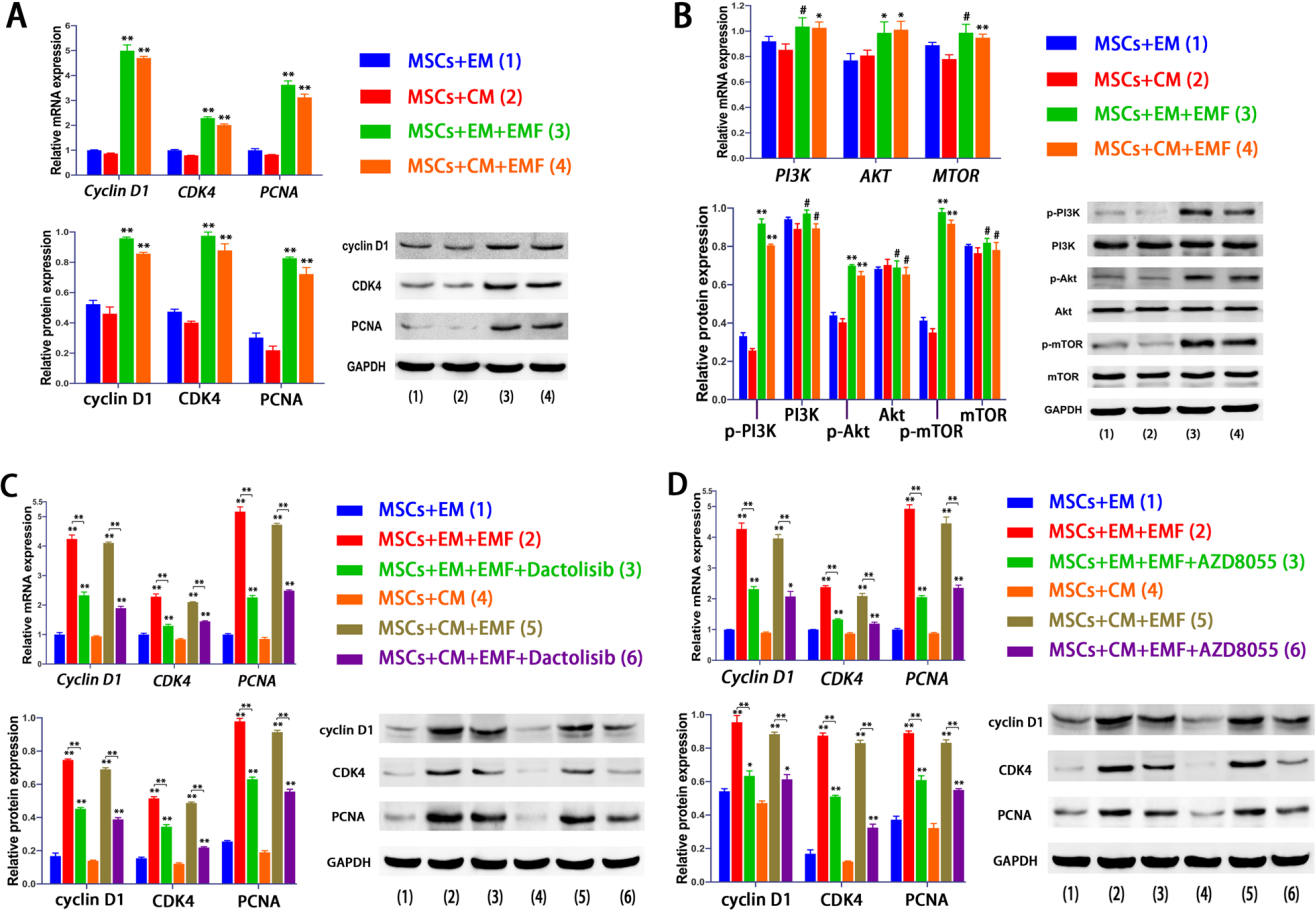
EMF enhanced expressions of proliferation-related genes and proteins partly by activating PI3K/Akt/mTOR pathway. **A** Expressions of Cyclin D1, CDK4, and PCNA were significantly increased in the presence of EMF. **B** Results of qRT-PCR and western blot showed that EMF promoted phosphorylation of PI3K, Akt, and mTOR proteins. **C** PI3K inhibitor (Dactolisib) could significantly reduce the increased expressions of Cyclin D1, CDK4, and PCNA induced by EMF both in gene and protein levels. **D** Inhibition of mTOR by AZD8055 showed results similar to those in **(C)**. Data are shown as mean ± standard deviation (*n* = 3). **#** indicates no significant difference, *p < 0.05, **p < 0.01, ***p < 0.001 compared to the group with or without EMF as indicated

As indicated in Fig. 3A, EMF significantly enhanced the expression of proliferation-related genes, including *Cyclin D1, CDK4,* and *PCNA*. The protein levels of these genes in EMF groups were also increased when compared to the control group (Fig. 3A). We explored the underlying mechanisms by which EMF promoted cell proliferation. When cells cultured in expansion medium, EMF group showed a higher level in the gene expression of *AKT* while no significant difference in PI3K and mTOR was observed, compared with the observations of the control group. However, in the case of using CM, the gene expressions of PI3K, AKT, and mTOR were increased remarkably in the presence of EMF exposure (Fig. 3B). However, there was no notable difference in the total protein expressions of PI3K, AKT, and mTOR between EMF groups and corresponding control groups (Fig. 3B). The phosphorylation of these three proteins was enhanced by EMF stimulation and the phosphorylated proteins, detected by western blot analysis, were significantly increased both in the culture with EM and CM (Fig. 3B).

To verify whether EMF affects cell proliferation through PI3K/AKT/mTOR pathway, we used Dactolisib (PI3K inhibitor) and AZD8055 (mTOR inhibitor) to observe the changes of proliferation-related gene and protein expressions, respectively. The results of PCR analysis revealed significantly lower gene expressions of *Cyclin D1, CDK4,* and *PCNA* by PI3K inhibition in comparison with EMF group (Fig. 3C). The expression levels of these proteins were partly suppressed by addition of Dactolisib, compared with EMF stimulation (Fig. 3C). The similar results were also demonstrated when mTOR inhibition by AZD8055 (Fig. 3D).

### EMF enhanced chondrogenesis of BMSCs partly by activating Wnt1/LRP6/β-catenin pathway

**Fig. 4:**
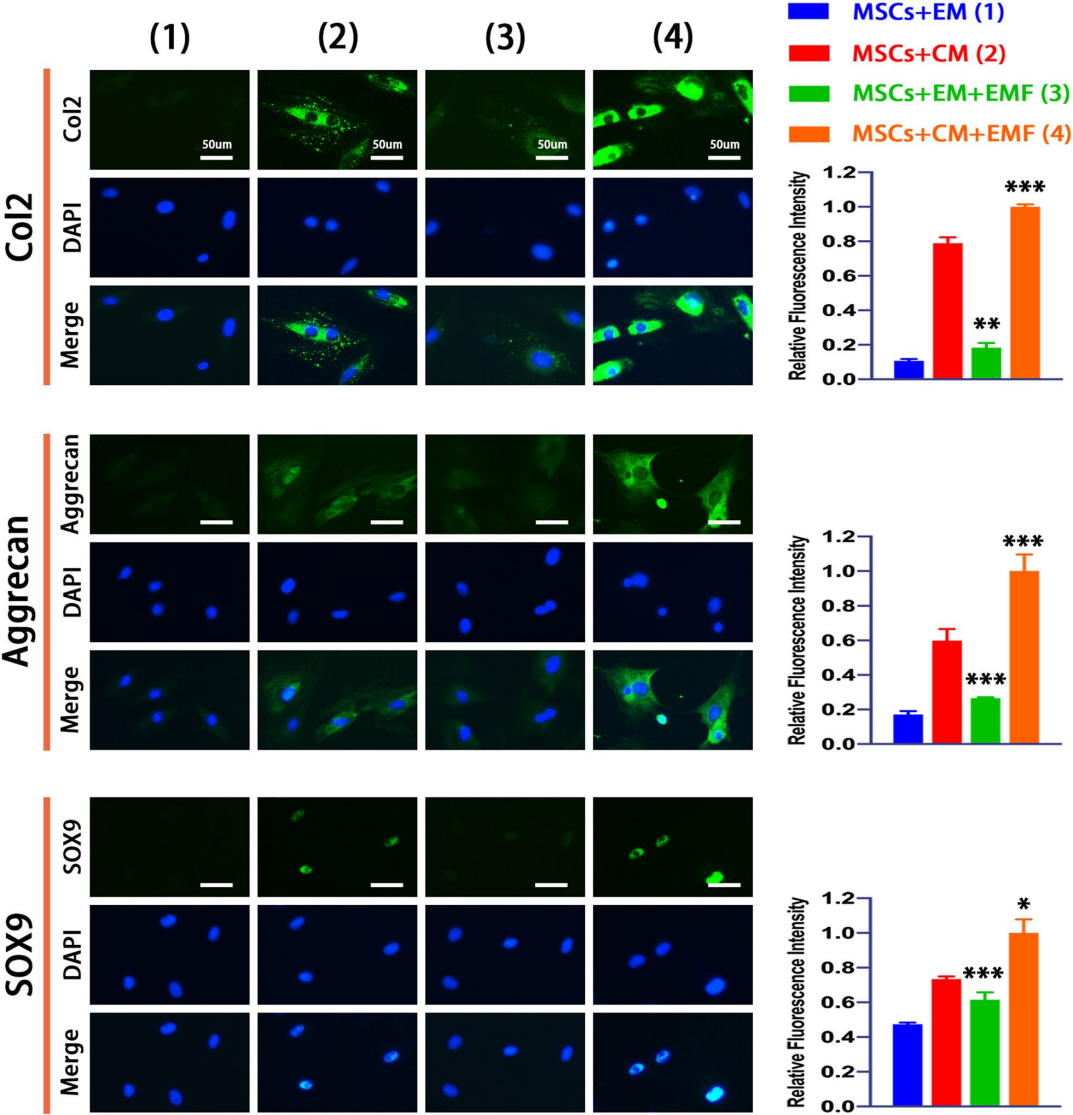
Immunofluorescence analysis of chondrogenesis-associated proteins. Immunofluorescence labeling and quantitative analysis indicate that chondrogenic medium dramatically improved expressions of chondrogenesis-associated proteins (including Col2, Aggrecan, and SOX9) and EMF could further enhance their intensities. Data are shown as mean ± standard deviation (*n* = 5). **#** indicates no significant difference, *p < 0.05, **p < 0.01, ***p < 0.001 compared to group without EMF

**Fig. 5:**
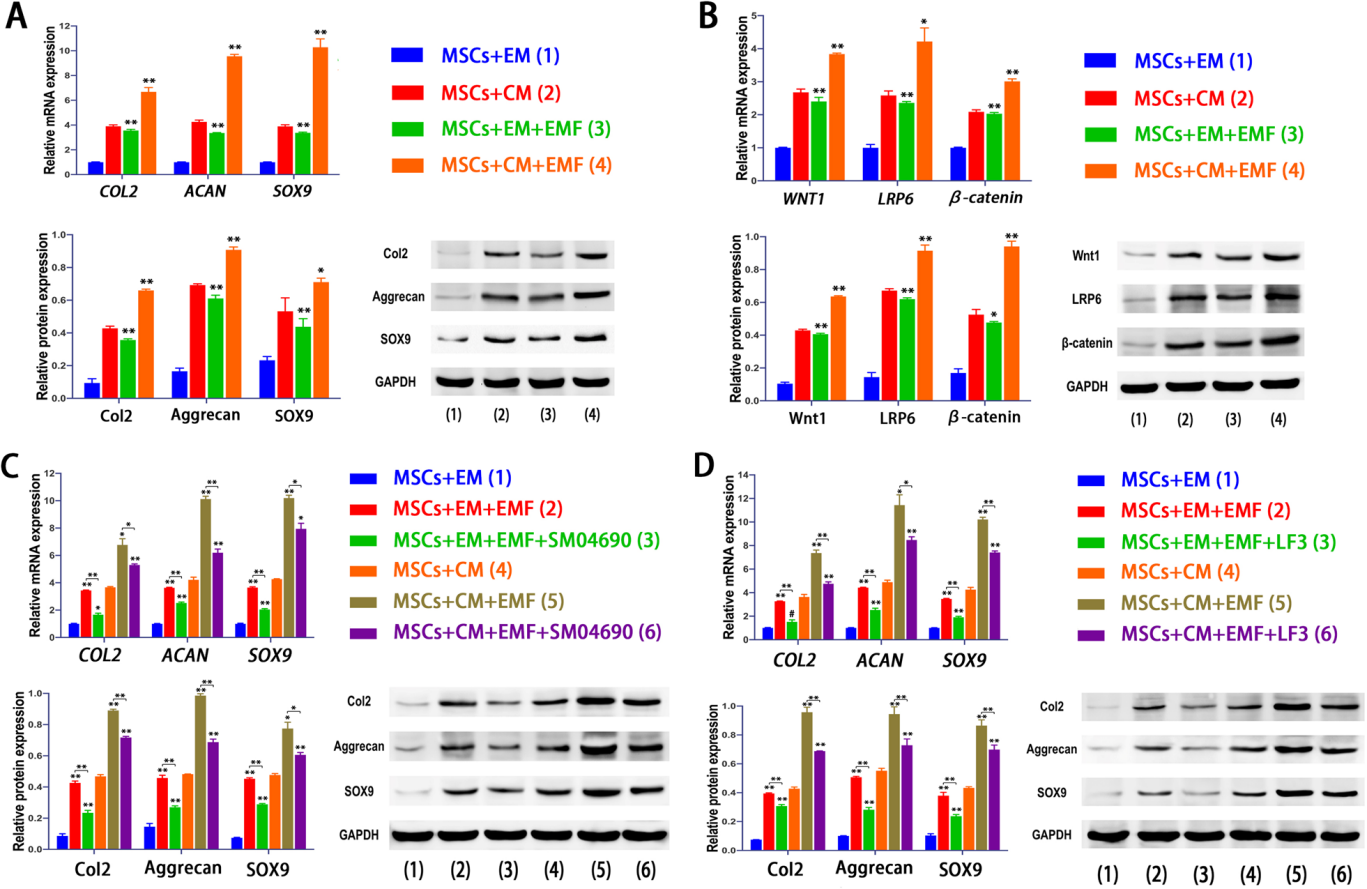
EMF enhanced chondrogenesis partly by activating Wnt1/LRP6/β-catenin pathway. **A** Expressions of Col2, Aggrecan, and SOX9 were significantly increased in the presence of EMF. **B** Results of qRT-PCR and western blot show that EMF activates the Wnt1/LRP6/β-catenin signaling pathway. **C** Wnt1 inhibitor (SM04960) significantly reduced the increased expressions of Col2, Aggrecan, and SOX9 induced by EMF both in gene and protein levels. **D** Inhibition of β-catenin by LF3 shows results similar to those in **(C)**. Data are shown as mean ± standard deviation (*n* = 3). **#** indicates no significant difference, *p < 0.05, **p < 0.01, ***p < 0.001 compared to group without EMF or the group as indicated

First, we analyzed the distribution and expression of chondrogenesis-related proteins (including Col2, Aggrecan, and SOX9) by immunofluorescence after EMF exposure. Regarding to the distribution, the images indicted that SOX9 was expressed almost entirely within nucleus, while Col2 and Aggrecan were distributed both in the nucleus and cytoplasm. Notably, the expressions of these proteins were all significantly strengthened by EMF according to the analysis of fluorescence intensity (Fig. 4). Besides, we also detected the gene expressions of *COL2, ACAN,* and *SOX9* by PCR. The EMF groups showed remarkably higher levels of these genes with a significant difference in comparison to control groups. The results of the western blot also revealed a remarkable increase in protein expressions (Fig. 5A).

Moreover, we investigated the potential mechanisms through which EMF played its prochondrogenic role in BMSCs. Both in EM and CM, the gene expression of *WNT1, LRP6,* and *β-catenin* in EMF groups were significantly higher than control groups. The analysis of protein expressions of Wnt1, LRP6, and β-catenin by western blot revealed similar trends (Fig. 5B). Then, the inhibitors of Wnt1 (SM04960) and β-catenin (LF3) were applied to demonstrate that EMF could promote chondrogenic differentiation of BMSCs by activating the Wnt1/LRP6/β-catenin signaling pathway. The inhibition of Wnt1 could partially downregulate the increased gene expressions, which were induced by EMF. The protein expressions of Col2, Aggrecan, and SOX9 were also reduced dramatically by SM04960 (Fig. 5C). When using β-catenin inhibitor (LF3), the lower gene expressions of *COL2, ACAN,* and *SOX9* were observed with statistically significance compared with EMF groups. Further, the protein expressions of Col2, Aggrecan, and SOX9 suggested a significant downregulation when inhibiting β-catenin with compared to EMF groups (Fig. 5D).

### EMF stimulation decreased stemness of BMSCs

**Fig. 6:**
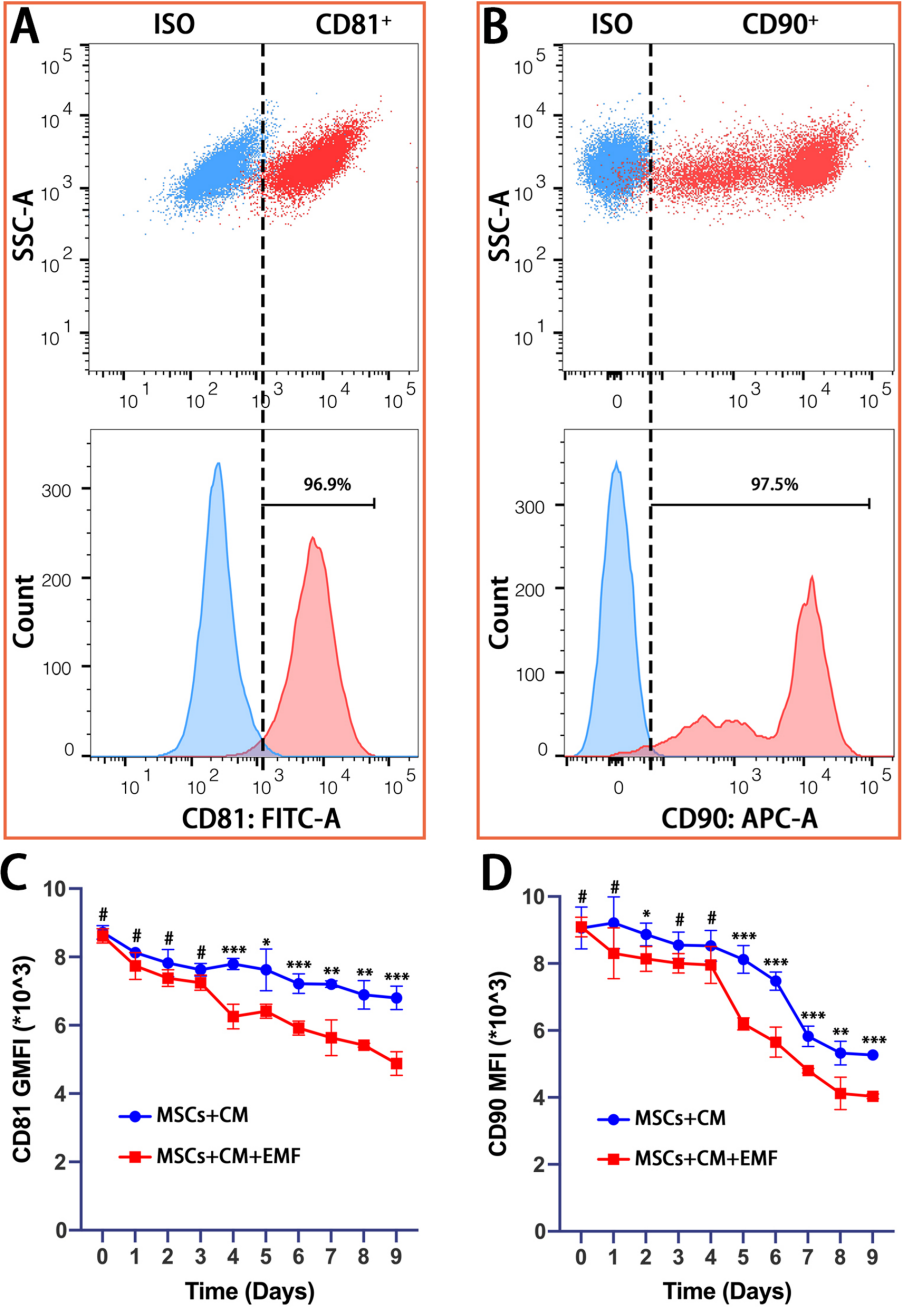
Identification of BMSCs from rabbits based on two surface markers (CD81 and CD90) and their changes for average fluorescence intensity with time by flow cytometry analysis. **A, B** Results show that 96.9% and 97.5% of cells positively express CD81 and CD90, respectively. **C, D** In the culture with chondrogenic medium, the fluorescence intensities of CD81 and CD90 are remarkably decreased in the presence of EMF for four and five days, respectively. Data are shown as mean ± standard deviation (*n* = 5). **#** indicates no significant difference, *p < 0.05, **p < 0.01, ***p < 0.001 compared to group without EMF

As reported by Tan et al. [53], rabbit BMSCs positively expressed several surface markers, including CD81 and CD90. We used these two markers to further identify the isolated BMSCs and the results of flow cytometry showed that 96.9% and 97.5% of cells positively expressed CD81 (Fig. 6A) and CD90 (Fig. 6B), respectively. Further, the average fluorescence intensities of CD81 (Fig. 6C) and CD90 (Fig. 6D) were both gradually reduced with time in the culture with CM. A remarkable decrease in CD81 fluorescence intensity was observed when BMSCs were stimulated by EMF for four days, and the decrease in CD90 for five days. Although during chondrogenesis induction over a period of nine days, the number of cells positively expressing CD81 or CD90 was approximately the same. The peak fluorescence intensities were both decreased with time. Furthermore, the lower intensity in EMF group indicated that EMF could accelerate the peak fluorescence intensity of CD81 and CD90 to shift to the left.

### EMF promoted osteochondral repair in a rabbit osteochondral defect model

**Fig. 7:**
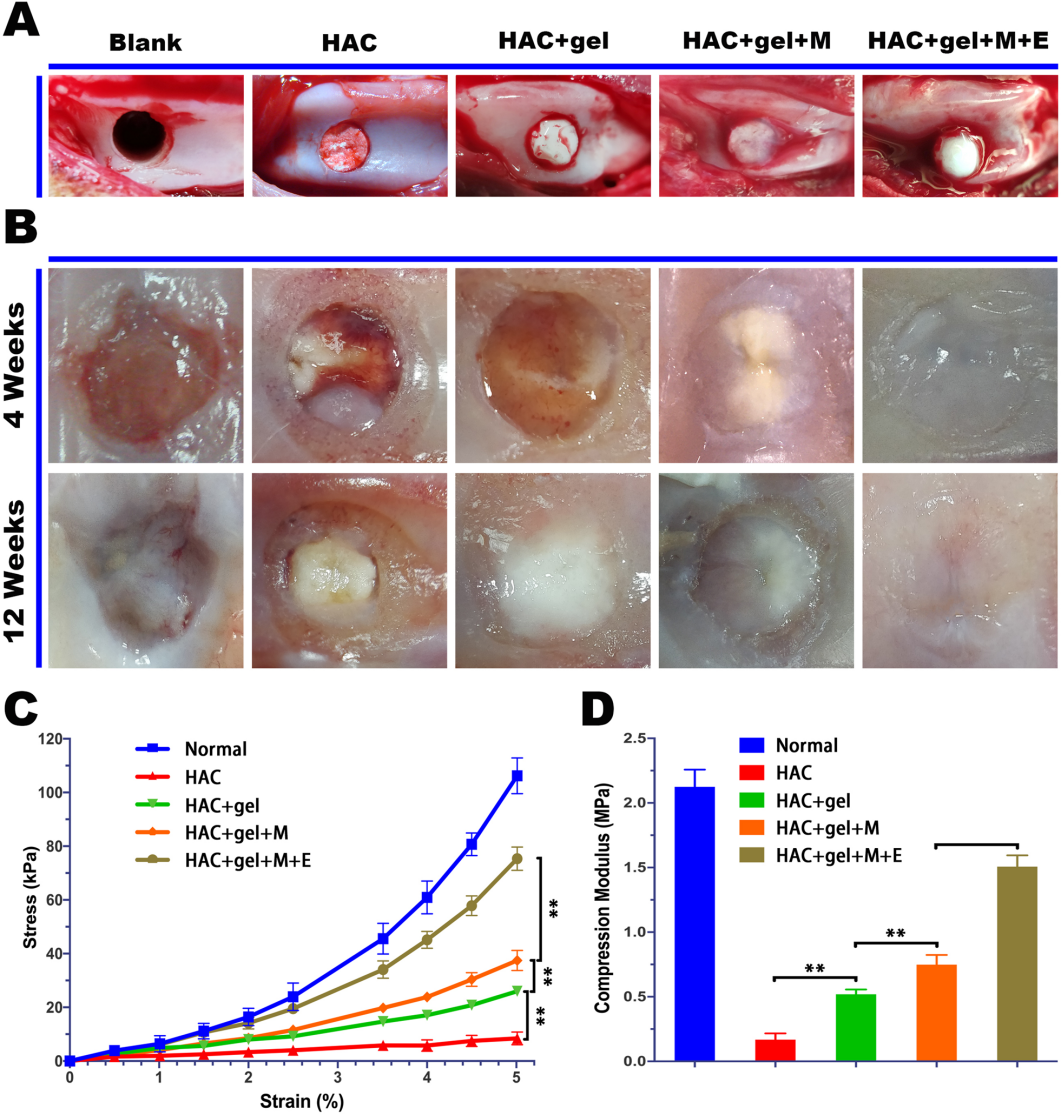
Morphological and mechanical evaluation of regenerative cartilage. **A** Representations of osteochondral defects with different treatments. **B** Macroscopic observation show that osteochondral repair was improved with time from 4 to 12 weeks post implantation. Particularly, the smooth neocartilage with similar appearance to adjacent native tissue was found in EMF group. **C** Strain–stress curve of regenerative tissue with representative points at intervals of 0.5% strain. **D** The compression modulus was calculated at strain of 5% and the highest modulus in EMF group suggested excellent mechanical properties. Data are shown as mean ± standard deviation (*n* = 5). **#** indicates no significant difference, *p < 0.05, **p < 0.01, ***p < 0.001 compared to the group as indicated

A rabbit model (Fig. 7A) with a critical-sized defect was used to assess the effect of EMF on osteochondral repair in vivo. As shown in (Fig. 7B), there was no obvious repair in the Blank group, and only a small amount of chondroid new-formed tissue was found around scaffold in HAC and HAC + gel groups. With regard to HAC + gel + MSCs group, the defect was filled with more cartilage-like tissue, although it was less transparent than that in EMF group. From 4 to 12 weeks after implantation, the defect repair was significantly enhanced in last four groups, while degradation was observed at the defect site in Blank group. Around the edge of defects, regenerated tissues were found both in HAC and HAC + gel groups. Besides, the HAC + gel group indicated a thin layer of cartilaginous tissue covering the defect hole. When BMSCs were planted within the thermogel, the new-formed tissue was remarkably increased. However, a clear boundary between the native and new tissue suggested an incomplete repair with poor lateral integration. Notably, this boundary was almost eliminated by application of EMF, and the repaired tissue showed a similar appearance to native hyaline cartilage.

### EMF stimulation increased mechanical strength of regenerated osteochondral tissue

We recorded the data of axial deformation and corresponding pressure and plotted the strain–stress curve based on representative points (Fig. 7C). Evidently, the EMF group showed a lowest strain under the same stress, which indicated the new-formed tissue in the EMF group possessed a satisfactory mechanical property with highest strength. Young’s modulus was also calculated at 5% axial strain in each group (Fig. 7D). The normal osteochondral tissue had a modulus above 2 MPa. The modulus of repaired tissue in EMF group was significantly higher than in the other three groups. These results suggested that EMF could significantly improve the biomechanical properties of new-formed osteochondral tissue.

### EMF treatment increased bone mass of both trabecular and subchondral bone in osteochondral repair

**Fig. 8:**
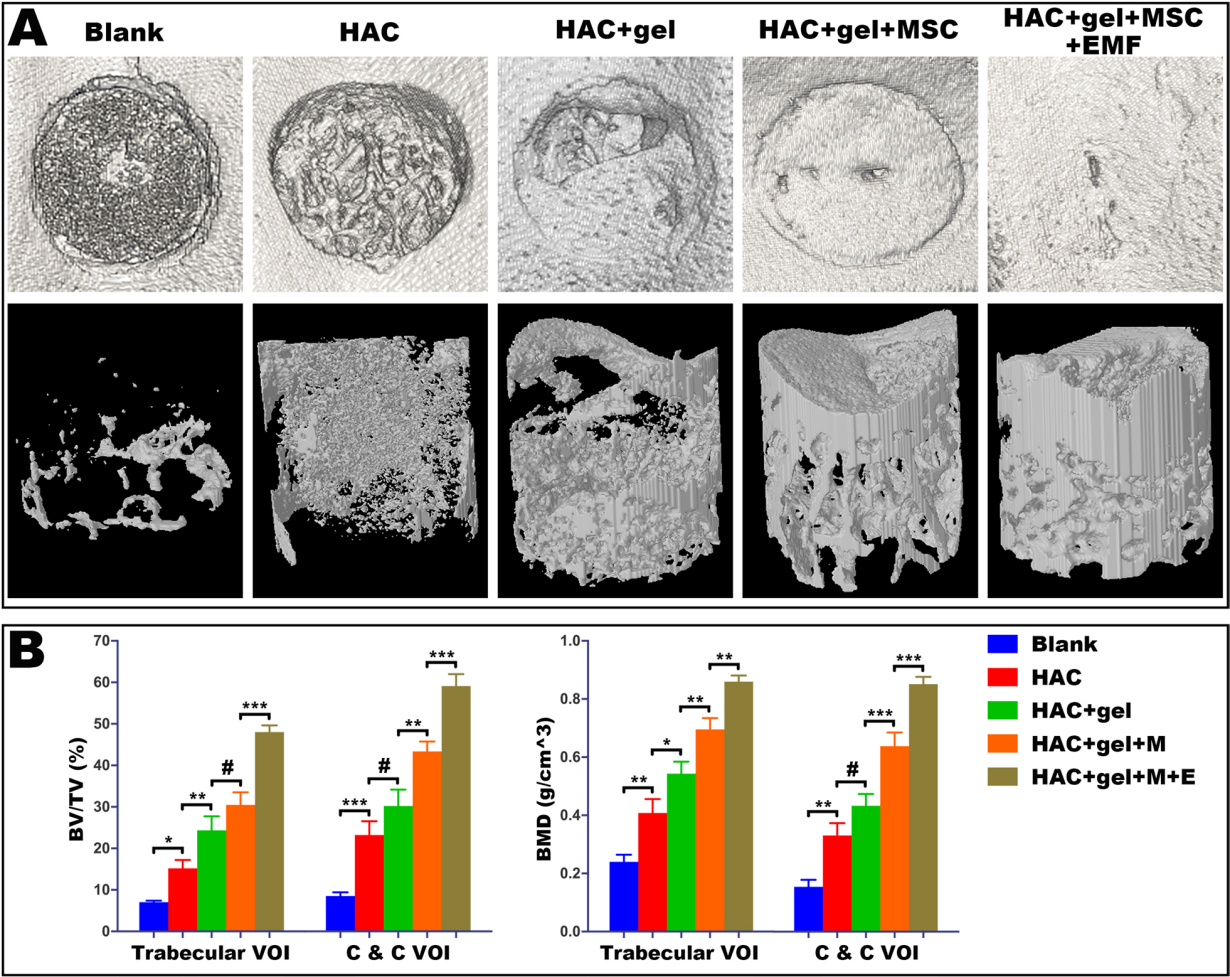
Micro-CT analysis for subchondral bone regeneration. **A** Reconstructed images of surfaces (first line) and defect area (second line) show improved repair incrementally in groups from left to right. **B** Quantitative analysis of micro-CT data further confirm that bone volume per total volume (BV/TV) and bone mineral density (BMD) were both incrementally increased in groups from left to right, in terms of trabecular volume of interest (VOI) and cartilage and cortical (C&C) VOI. The values of BV/TV and BMD were highest in the EMF group. Data are shown as mean ± standard deviation (*n* = 5). **#** indicates no significant difference, *p < 0.05, **p < 0.01, ***p < 0.001 compared to the group as indicated

Micro-CT was further performed to characterize regenerated tissue by reconstruction of 3D images and analysis of statistical data. For the Blank group, there was little self-repair bone with a large cavity. In terms of defect surface, images showed that regenerative bone in EMF group was the densest with good integration to adjacent native tissue. The amount of new bone repaired by the cellular scaffold was also significantly increased compared with the cell-free groups. The largest amount of new bone was observed in the EMF group (Fig. 8A). The bone volume per total volume (BV/TV) was highest in the EMF group in both the trabecular volume of interest (VOI) and cartilage and cortical (C&C) VOI when compared to other groups. The same trend was also shown for BMD for both trabecular VOI and C&C VOI (Fig. 8B).

### EMF treatment promoted cartilage repair

**Fig. 9:**
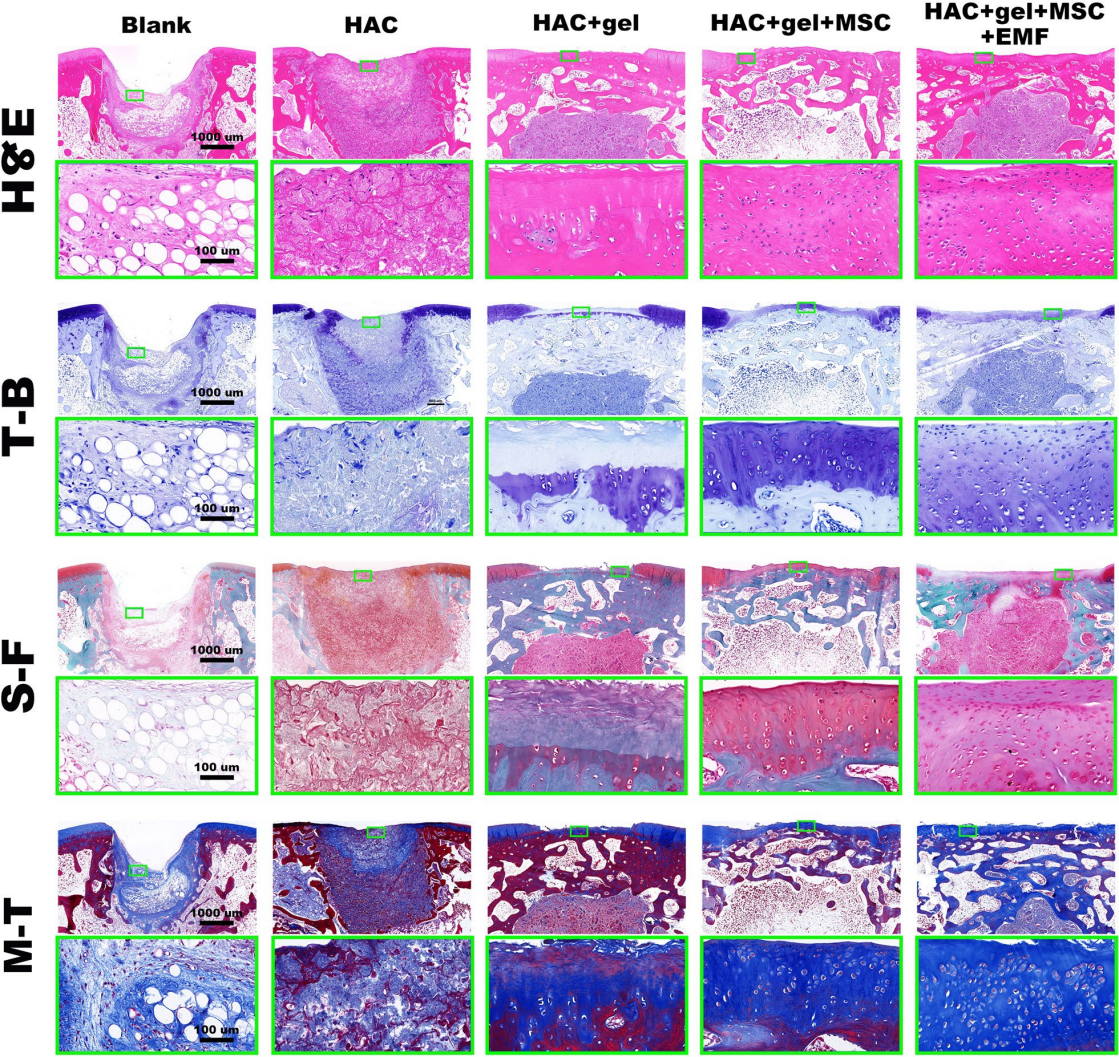
Histological analysis of newly formed tissue at 12 weeks post-surgery. The staining of hematoxylin and eosin (H&E), toluidine blue (T-B), and Safranin O-fast green (S-F) showed that there was obvious defect in the Blank group, little neocartilage in cell-free groups, and evident repair in cell-loaded groups. In particular, the EMF group had the thickest newly formed tissue in the cartilage layer with most chondrocytes and a well-integrated interface. Masson's Trichrome (M-T) staining also suggested enhanced subchondral bone regeneration and HAC scaffold degeneration in EMF group compared to other groups. The magnification of pictures are 3X for upper rows and 30X for lower rows

Histological staining was performed to confirm the effect of EMF on promoting osteochondral repair, especially for the repair of damaged cartilage (Fig. 9). In cell-loaded groups, a layer of new-formed cartilage was observed within the chondral defect region. Furthermore, staining of both toluidine blue (T-B) and safranin O-fast green (S-F) indicates that the new cartilage in HAC + gel + M + E group is significantly flatter and thicker with a better integration to adjacent native cartilage, compared to the HAC + gel + M group. We also found a pattern of chondrocytes arrangement within new cartilage similar to that of native cartilage in EMF group, which appeared to be more orderly and aligned.

**Fig. 10:**
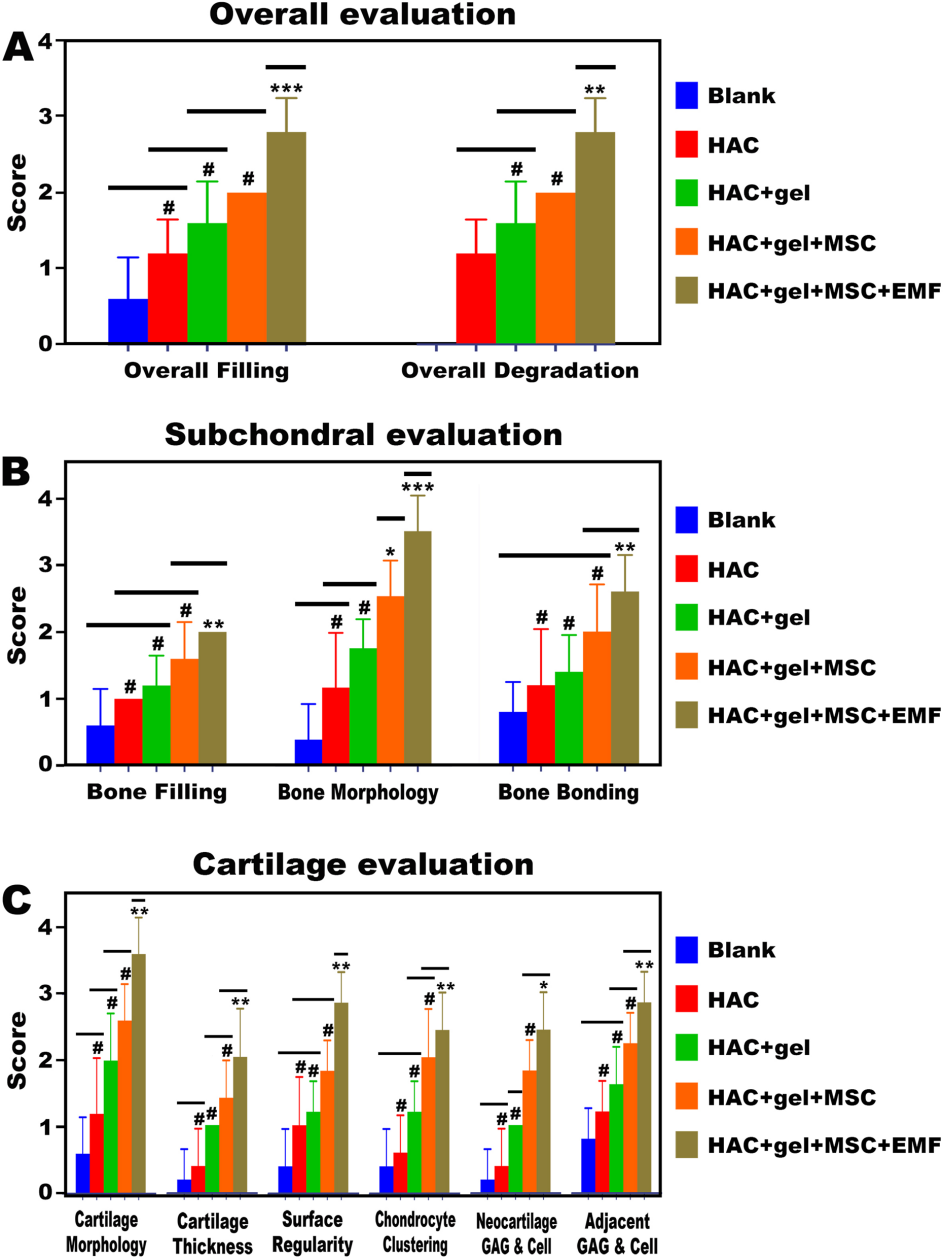
Histological scores for evaluation of osteochondral repair. **A** Scores for overall defect evaluation. **B** Scores for subchondral bone evaluation. **C** Scores for cartilage evaluation. Compared to the Blank group, scores for all parameters except bone morphology were significantly higher in thee EMF group. Data are shown as median (inter quartile range (IQR)) (*n* = 5). # indicates no significant difference, *p < 0.05, **p < 0.01, ***p < 0.001 compared to blank group. The bold horizontal indicates no significant difference among groups under its coverage

The histological staining was further assessed by using a quantitative scoring system. In terms of overall defect evaluation, the same scores for overall filling and overall degradation suggested there was a dynamic balance between tissue regeneration and scaffold degradation (Fig. 10A). Significant differences for both overall filling and degradation were found only in EMF group, but not in other three groups when compared to the Blank group. For subchondral bone evaluation, the scores of bone filling, bone morphology, and bone bonding were all highest in the EMF group (Fig. 10B). Although the bone morphology score in HAC + gel + M was also dramatically higher than that in Blank group. In terms of cartilage evaluation, all parameters remained the highest in EMF group and exhibited significant differences when compared with Blank group (Fig. 10C).

### EMF promoted expression of bone and cartilage repair makers in both protein and RNA level

**Fig. 11:**
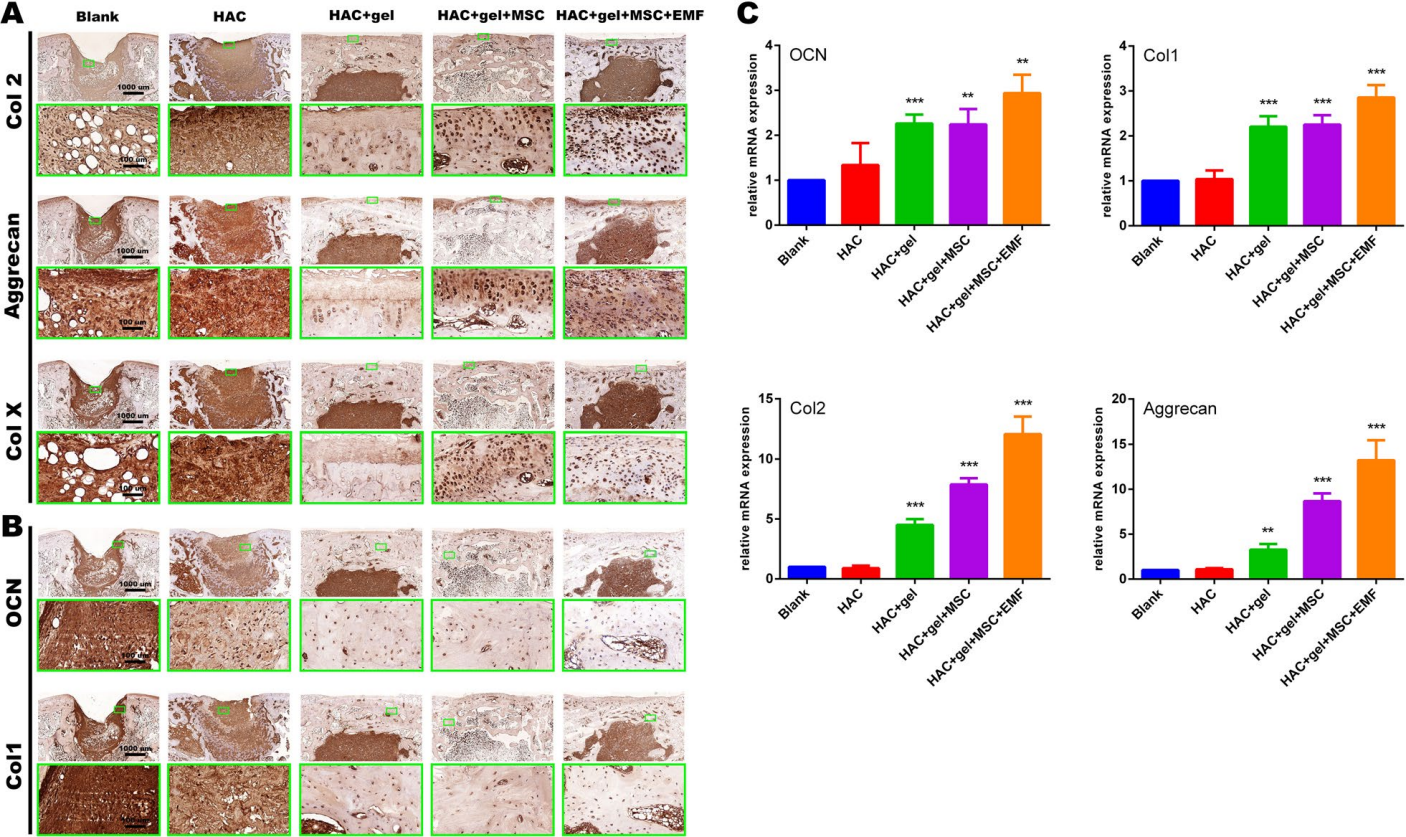
Immunohistochemical staining for evaluation of chondrogenesis- and osteogenesis-related proteins. **A** Positive staining of Col2, Aggrecan, and Col X was evident in cell-loaded groups, while there were almost no positive cells in the first three groups without BMSCs. The EMF group showed the strongest staining of Col2 and Aggrecan proteins. The Col X expression in EMF group was relatively lower compared to the “HAC + gel + MSC” group. **B** Osteocalcin (OCN) and Col1 were visualized in the last three groups, and their staining was remarkably stronger compared to other two groups. The number of cells positively expressing OCN or Col1 was relatively higher in the EMF group than those in “HAC + gel” and “HAC + gel + MSC” groups. (C) RNA expression of Col 2, Aggrecan, Col X, OCN and Col 1 in regenerated tissue was measured by RT-PCR. The HAC + gel + MSC + EMF group showed highest level of Col 2, Aggrecan, Col X, OCN and Col 1 relative RNA expression, and significantly higher than any other groups. The magnification of immunohistochemical staining pictures are 3X for upper rows and 30X for lower rows. Data are shown as mean ± standard deviation (*n* = 5). *p < 0.05, **p < 0.01, ***p < 0.001 compared to blank group

Several cartilage-specific proteins were stained by immunohistochemical methods to further evaluate the new cartilage (Fig. 11A). The positive staining regions were matched with those in T–B and S–F staining. In the last two groups with BMSCs, the staining of Col2 and Aggrecan were both positive, while there was almost no positive staining in cell-free groups. The stronger staining of these two proteins suggested an increase in the protein expression of the EMF group above the one in the HAC + gel + M group. The most positive chondrocytes within the chondral defect were observed in the EMF group, with respect to both Col2 and Aggrecan. The expression of Col X indicates the hypertrophy and calcification of chondrocytes. Col X was relatively reduced in EMF group compared to HAC + gel + M group. OCN and Col1 were also stained for evaluation of subchondral bone repair (Fig. 11B). The positive staining was negligible in the first two groups, while both proteins were evident in last three groups. We also extracted RNA from regenerated tissue and tested relative RNA levels of the above markers by RT-PCR. In accordance with the Immunohistochemical staining, the RT-PCR results showed that EMF treatment significantly increase the RNA expression of Col 2, Aggrecan, Col X, OCN and Col 1 (Fig. 11C). These results further indicate that EMF could enhance cartilage and subchondral bone repair.

## Discussion

Articular cartilage repair remains a significant challenge for researchers and clinicians, especially in the reconstruction of hyaline cartilage with proper biomechanical function [54, 55]. Compared to other methods of cartilage repair, such as bone marrow stimulation and autografts implantation, cartilage tissue engineering appears to be the most attractive therapy. With the development of new biomaterials and fabrication techniques, a variety of scaffolds have emerged with hierarchical structures, excellent mechanical properties, and biological performance [56, 57]. Such scaffolds provide a three-dimensional microenvironment for growth and migration of cells derived from diverse sources that have the potential to differentiate into chondrocytes [5]. Numerous scaffolds have been investigated for cartilage reconstruction, among which the polymeric materials attracted significant attention, primarily in forms of sponges, fibrous meshes, and hydrogels. The injectable thermosensitive hydrogels can easily fill the cartilage defect with irregular shape and be transplanted by a minimally invasive procedure. More importantly, the cells could be suspended homogenously within thermogels in a sol state and proliferate without loss of differentiation potential [58]. Hydrogels simulate physiological ECM surrounding chondrocytes and transmit external stress to cells encapsulated in gels. After sol-to-gel transition, hydrogels also obtain certain mechanical strength, although such mechanics are limited. In addition, the defect of cartilage is often accompanied by the damage of subchondral bone, hence the repair of subchondral bone is key to cartilage regeneration. We employed the HAC scaffold to repair the bone defects in the previous study. Thereby, a composite scaffold consisting of HAC and PLGA-PEG-PLGA thermogel was produced to repair the osteochondral defect in this study. The repair requirements for articular cartilage and subchondral bone are different owing to their distinct physiological features and functions. Our results show that it was feasible to reconstruct cartilage and subchondral bone by two types of materials, which were suitable for repairing cartilage and bone, respectively.

Biophysical stimulations exert significant effects on cellular behavior, and some have been applied in clinical practice [59]. In addition to chemical factors, physical signals also play a regulatory role in the development and regeneration of bone and cartilage. The Food and Drug Administration (FDA) has approved the use of pulsed electromagnetic fields (PEMFs) for the treatment of delayed union and nonunion of bone fractures. Although increasing evidence suggested that PEMF could be an alternative therapy for cartilage repair, clinical application of PEMF in joints is still debated and more research is needed [60]. A narrative review summarized the current research involving the effect of EMF on articular cartilage and potential application in joint diseases [38]. The stimulation of PEMF could increase intracellular calcium concentration by regulating calcium channels, which are required for the chondrogenic differentiation of MSCs [31]. When the parameters of EMF were tuned to the cyclotron resonance frequencies of calcium ions, Kavand et al. reported an enhanced efficacy of chondrogenesis [33]. Recently, one study showed that the MSC-derived conditioned medium post EMF exposure was also capable to promote cartilage regeneration and demonstrated that EMF could regulate the paracrine function of MSCs [36]. Previous studies have shown that Wnt1/β-catenin signaling pathway is regulated by EMF in osteogenesis and bone metabolism [61, 62]. Based on our results in this study, we further verified that EMF could also facilitate chondrogenic differentiation and contribute to improving cartilage repair by activating the Wnt1/LRP6/β-catenin pathway.

The cell proliferation as well as ECM synthesis are both crucial in the cartilage regeneration. The positive effect of EMF on cell proliferation has been observed in various cell types, such as MSCs [63], chondrocytes [64], and osteoblasts [65]. Consistently with previous results, our study further demonstrated that EMF could promote proliferation of BMSCs by upregulating PI3K/Akt/mTOR pathway. Nevertheless, some studies indicate that EMF has no effects on cell proliferation[66, 67].The cell types and EMF parameters may together account for these inconsistent results. More importantly, the cartilage defects would cause joint inflammation, which in turn exacerbates cartilage deterioration. Breaking this vicious cycle will significantly contribute to cartilage repair. Numerous studies have elaborated on the possible mechanisms that EMF alleviates inflammatory response. For instance, the anti-inflammatory effect of EMF appeared to be mediated by increasing adenosine receptors like A_2_A and A_3_ [68–70]. In mice with osteoarthritis (OA), EMF inhibited the expression of inflammatory cytokines, including IL‐1β, ADAMTS4, and MMP13 [71]. Moreover, the effects of EMF on inflammation and underlying mechanisms have been systematically reviewed in terms of OA [72] or RA [39]. Although the application of EMF to treat OA or RA must be verified by more clinical trials with long-term follow-up, it is also one of the possible mechanisms by which EMF enhances cartilage repair.

For tissue repair, there have been many preclinical and clinical studies on the role of EMF in bone repair, while only few addressed cartilage regeneration [37, 73]. A review depicted the potential benefits of EMF in cartilage repair [70]. To develop therapeutic strategy combined with EMF, it is necessary to optimize EMF parameters, such as the waveform, field direction, intensity, frequency, and time of exposure. However, the combinations of above parameters are theoretically innumerable, and there is no experiment design that can cover all these parameters. Thus, the community of EMF research must focus on unveiling the underlying laws and mechanisms, by which EMF exerts its biological effects. Moreover, the type of recipient cells or tissues stimulated by EMF is another essential factor that affects the quality of tissue repair [74]. With the advances of novel physical stimulus modalities, the application of EMF in medicine will be further broadened, and this effect will be reinforced. Several studies have introduced magnetic beads that can feel the force of EMF to biological culture system. Song et al. reported that the magnetic beads could promote cell proliferation driven by EMF. TGF-β-immobilized magnetic beads successfully induced MSCs to generate cartilage in vitro under magnetic forces [75, 76]. Moreover, EMF can operate without direct contact and thus enable extracorporeal control.

The articular cartilage defect is a common medical problem that severely affects the life quality of patients. The therapeutical strategies currently used in clinical practice relief associated symptoms and yield acceptable outcomes in the short term. However, the repaired cartilage was found to degrade during a long period of follow-up, as new-formed tissue was often fibrocartilage rather than hyaline-like cartilage [77]. Thus, the ambitious goal of cartilage repair is to regenerate hyaline cartilage with proper mechanical function and prevent long-term degradation. To the best of our knowledge, our study was the first to demonstrate that sinusoidal EMF can significantly improve cartilage regeneration when combined with tissue engineering. We also explored the underlying mechanisms and found that PI3K/AKT/mTOR and Wnt1/LRP6/β-catenin signaling pathways mediated the transduction of biological effects of EMF for cell proliferation and differentiation, respectively. Furthermore, the lateral integration of repair cartilage with adjacent native tissue was improved, which may further contribute to the effect of EMF on cartilage repair. Furthermore, EMF could also enhance bone repair, which has been extensively studied. In short, EMF plays an essential role in the repair of osteochondral defects, and more research is required to explore optimal parameters and underlying mechanisms, especially for cartilage regeneration. These encouraging results from our study can pave the way for the application of biophysical stimuli in clinical cartilage repair.

## Conclusion

A composite scaffold was constructed using a HAC and PLGA–PEG–PLGA thermogel, which matched with cartilage and subchondral layers in osteochondral defects, respectively. The BMSCs encapsulated in the thermogel were stimulated by EMF. Results from in vitro experiments suggested that EMF could promote proliferation and chondrogenic differentiation of BMSCs by activating the PI3K/AKT/mTOR and Wnt1/LRP6/β-catenin pathways, respectively. In vivo evaluation further confirmed that EMF could enhance repair of osteochondral defects, especially cartilage repair, combined with tissue engineering in rabbits. Therefore, this study significantly advances the potential application of EMF or even other biophysical stimuli towards tissue regeneration.

## Supplementary Information

**Additional file 1.** The sequences of primers used in this study.

**Additional file 2.** Histological scores for all groups with different treatments for osteochondral defect in rabbits 12 weeks post-surgery.

## Data Availability

The datasets used and/or analyzed during the current study are available from the corresponding author on reasonable request.

## Abbreviations

BMD: Bone mineral density
BMSC: Bone marrow mesenchymal stem cells
CM: Chondrogenic medium
EM: Expansion medium
EMF: Electromagnetic field
H&E: Hematoxylin and eosin
PEMF: Pulsed electromagnetic fields
VOI: Volume of interest
