# Supplementary material for: Hydrogel-hydroxyapatite-monomeric collagen type-I scaffold with low-frequency electromagnetic field treatment enhances osteochondral repair in rabbits
Source: Stem Cell Res Ther. 2021 Nov 13;12:572. doi: 10.1186/s13287-021-02638-6 (PMC8590294; doi:10.1186/s13287-021-02638-6)
Supplement: Supplementary file 1 — Additional file 1. The sequences of primers used in this study. [file 13287_2021_2638_MOESM1_ESM.docx]

**Table S1.** The sequences of primers used in this study.

| ***Gene*** | **Forward (5’-3’)** | **Reverse (5’-3’)** |
| --- | --- | --- |
| *COL2* | GCAACAGCAGGTTCACTTACAC | AGGAAGGGCAAACGAGATGG |
| *ACAN* | CAACGCATTGAGTGTGAGCATC | CAGCACCACCTCCTTGTCC |
| SOX9 | CTGGAGACTGCTGAACGAGAG | TCCGCCTGCCCATTCTTC |
| *Cyclin D1* | ACCCTGGTTCCGCCTTTG | TTCACAGCCGCCTCATCC |
| *CDK4* | CCACCGAGACCTGAAGCC | ACCAGAGCGTCACAACCAC |
| *PCNA* | AGATAATGCGGACACCTTGGC | GCTGAGGTCTCGGCATATACG |
| *WNT1* | TCTTCGGCAAGATCGTCAACC | GATGGAACCCTCGGAGCAG |
| *LRP6* | CTGTGGCTTGGCGTTGTG | TTATCCTGGCAGTTGGCATCTC |
| *β-catenin* | GACACCAGGAAGCGGAGATG | AACTGAACTAGGCGTGGAATGG |
| *PI3K* | CTGATCTTCCTCGTGCTGCTC | GTGGACAGTGTTCCTCCTTAGC |
| *AKT* | AGAAGGTTGGGTTCAGAAGAGG | ATGTGTTTGGCTTTGGTCGTTC |
| *MTOR* | GCGAGATGAGTCAGGAAGAGTC | CCGATGAGGCTGGCTATGG |
| *GAPDH* | CCACTTTGTGAAGCTCATTTCCT | TCGTCCTCCTCTGGTGCTCT |
