# Supplementary material for: Hydrogel-hydroxyapatite-monomeric collagen type-I scaffold with low-frequency electromagnetic field treatment enhances osteochondral repair in rabbits
Source: Stem Cell Res Ther. 2021 Nov 13;12:572. doi: 10.1186/s13287-021-02638-6 (PMC8590294; doi:10.1186/s13287-021-02638-6)
Supplement: Supplementary file 2 — Additional file 2. Histological scores for all groups with different treatments for osteochondral defect in rabbits 12 weeks post-surgery. [file 13287_2021_2638_MOESM2_ESM.doc]

**Table S2.** Histological scores for all groups with different treatments for osteochondral defect in rabbits 12 weeks post-surgery.

| **Histological Parameter** | **Scores, Median (IQR)** | | | | |
| --- | --- | --- | --- | --- | --- |
|  | **Blank** | **HAC** | **HAC+gel** | **HAC+gel+M** | **HAC+gel+M+E** |
| ***Overall defect evaluation*** |  |  |  |  |  |
| 1. Percent filling with newly formed tissue | 1 (0-1) | 1 (1-1.5) | 2 (1-2) | 2 (2-2) | 3 (2.5-3) |
| 2. Percent degradation of the implant | NA | 1 (1-1.5) | 2 (1-2) | 2 (2-2) | 3 (2.5-3) |
| ***Subchondral bone evaluation*** |  |  |  |  |  |
| 3. Percent filling with newly formed tissue | 1 (0-1) | 1 (1-1) | 1 (1-1.5) | 2 (1-2) | 2 (2-2) |
| 4. Subchondral bone morphology | 0 (0-1) | 1 (0.5-2) | 2 (1.5-2) | 3 (2-3) | 4 (3-4) |
| 5. Extent of new tissue bonding with adjacent bone | 1 (0.5-1) | 1 (0.5-2) | 1 (1-2) | 2 (1.5-2.5) | 3 (2-3) |
| ***Cartilage evaluation*** |  |  |  |  |  |
| 6. Morphology of newly formed surface tissue | 1 (0-1) | 1 (0.5-2) | 2 (1.5-2.5) | 3 (2-3) | 4 (3-4) |
| 7. Thickness of newly formed cartilage | 0 (0-0.5) | 0 (0-1) | 1 (1-1) | 1 (1-2) | 2 (1.5-2.5) |
| 8. Joint surface regularity | 0 (0-1) | 1 (0.5-1.5) | 1 (1-1.5) | 2 (1.5-2) | 3 (2.5-3) |
| 9. Chondrocyte clustering | 0 (0-1) | 1 (0-1) | 1 (1-1.5) | 2 (1.5-2.5) | 2 (2-3) |
| 10. Chondrocyte and GAG content of neocartilage | 0 (0-0.5) | 0 (0-1) | 1 (1-1) | 2 (1.5-2) | 2 (2-3) |
| 11. Chondrocyte and GAG content of adjacent cartilage | 1 (0.5-1) | 1 (1-1.5) | 2 (1-2) | 2 (2-2.5) | 3 (2.5-3) |

IQR, Inter Quartile Range; HAC, Hydroxyapatite and Collagen I; M, BMSCs; E, Electromagnetic Fields (EMF); GAG, Glycosaminoglycan;

NA, not available.
